# Supplementary material for: The interactome of intact mitochondria by cross-linking mass spectrometry provides evidence for coexisting respiratory supercomplexes
Source: Mol Cell Proteomics. 2017 Dec 8;17(2):216–32. doi: 10.1074/mcp.RA117.000470 (PMC5795388; doi:10.1074/mcp.RA117.000470)
Supplement: Supplemental Data [file supp_RA117.000470_133922_0_supp_23978_fzffwf.zip › spectra_annotation/mito_DR_spectra_annotation/203-2-2-1-4-1.pdf]

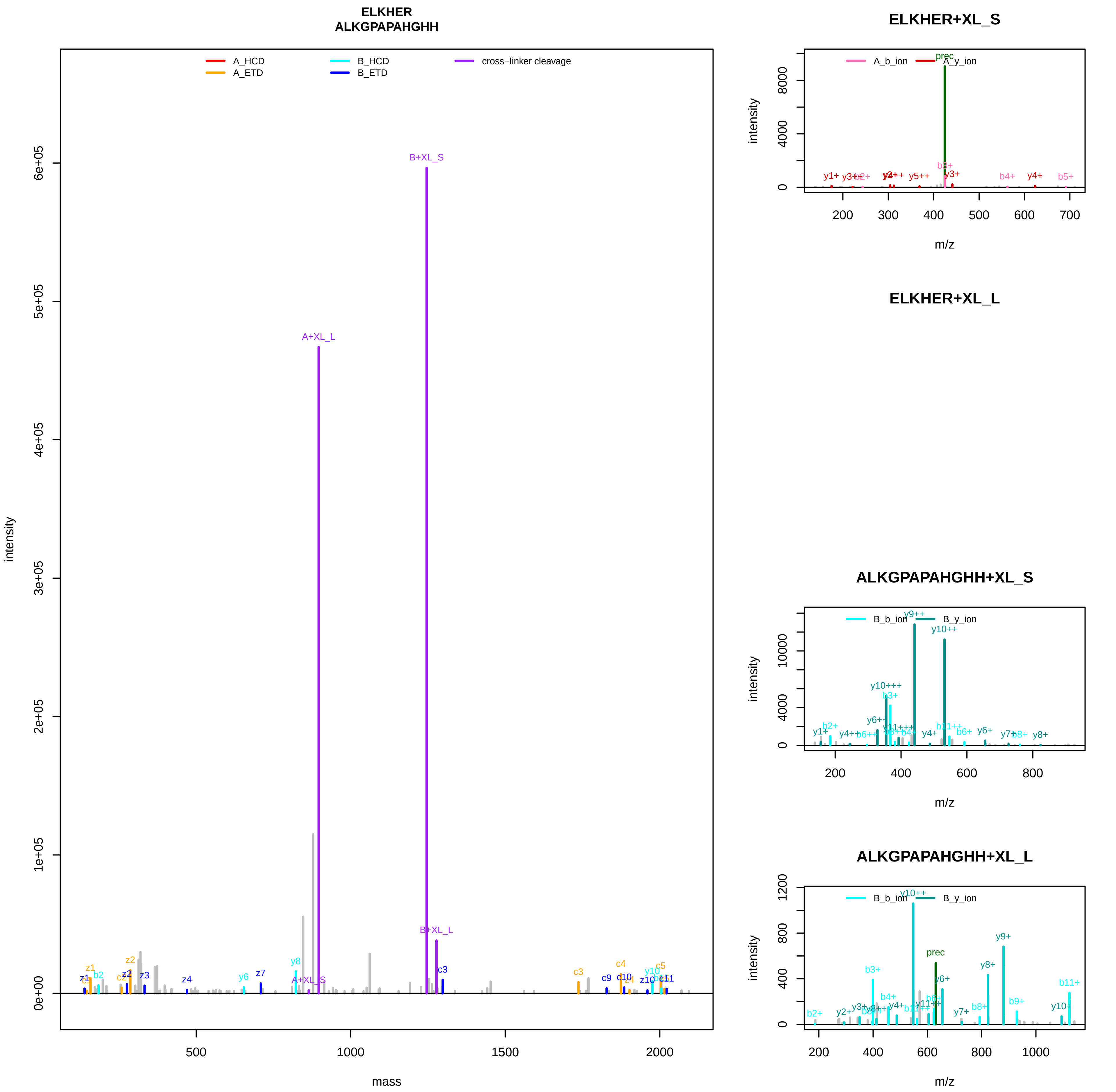

ELKHER
ALKGPAPAHGHH
A_HCD
A_ETD
B_HCD
B_ETD
cross−linker cleavage
B+XL_S
A+XL_L
B+XL_L
A+XL_S
intensity
mass
0e+00
1e+05
2e+05
3e+05
4e+05
5e+05
6e+05
500
1000
1500
2000
z1
b2
z2
z3
z4
y6
z7
y8
c3
c9
c10
c4
z10
y10
c5
c11
ELKHER+XL_S
A_b_ion
A_y_ion
prec
y1+
y5++
b3+
y3+
b4+
y4+
b5+
0
4000
8000
200
300
400
500
600
700
m/z
ELKHER+XL_L
ALKGPAPAHGHH+XL_S
B_b_ion
B_y_ion
y9++
y10++
y10+++
b3+
y6++
b2+
y1+
y4++
b6++
y4+
b11++
b6+
y6+
y7+
b8+
y8+
0
4000
10000
200
400
600
800
ALKGPAPAHGHH+XL_L
y10++
prec
y9+
y8+
b3+
b4+
y6+
b9+
b11+
y10+
y7+
b8+
b2+
y2+
y3+
y4+
0
400
800
1200
200
400
600
800
1000
